# Supplementary material for: Highly Strong and Damage-Resistant Natural Rubber Membrane via Self-Assembly and Construction of Double Network
Source: Membranes (Basel). 2022 Sep 26;12(10):933. doi: 10.3390/membranes12100933 (PMC9611818; doi:10.3390/membranes12100933)
Supplement: Supplementary file 1 [file membranes-12-00933-s001.zip › membranes-1900984-supplementary.pdf]

membranes

Supplementary Materials

Highly Strong and Damage-Resistant Natural Rubber Membrane via Self-Assembly and Construction of Double Network

Heliang Wang [1,2], Fanrong Meng [1], Mingyuan Yi [2], Lin Fang [2], Zhifen Wang [1,2,*] and Shoujuan Wang [1,*]

1 State Key Laboratory of Biobased Material and Green Papermaking, Qilu University of Technology (Shandong Academy of Sciences), Jinan 250353, China
2 College of Materials Science and Engineering, Hainan University, Haikou 570228, China
* Correspondence: 990385@hainanu.edu.cn (Z.W.); wshj@qlu.edu.cn (S.W.)

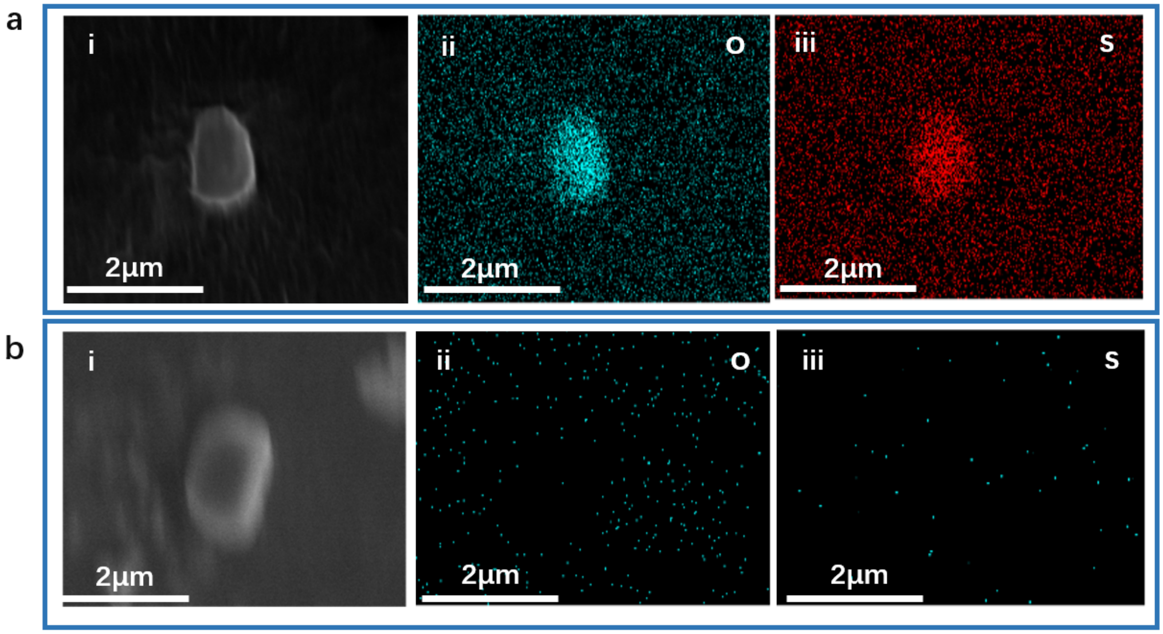

Figure S1. Element scan image of element distribution of (**a**) MSLS/NRL and (**b**) NRL latex particles.

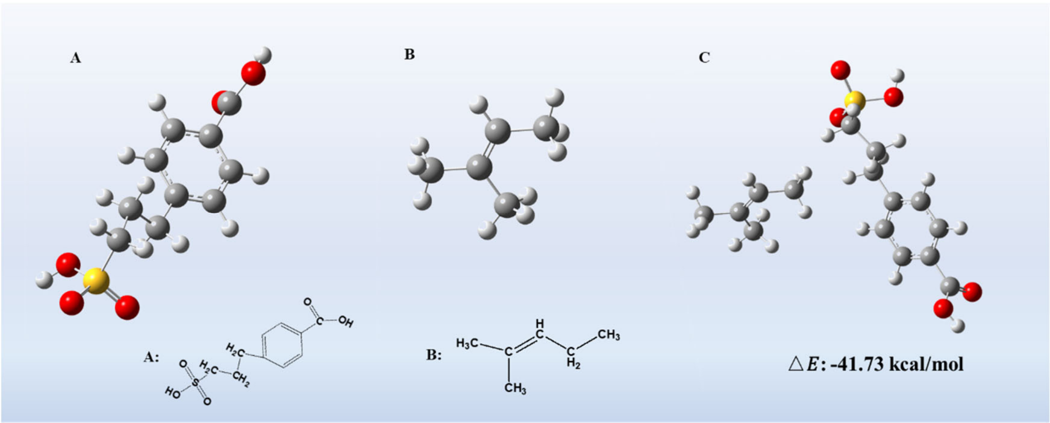

Figure S2. Density functional theory (DFT) calculation evaluating interaction energy between the MLSL and natural rubber.

To further study the core-shell formation mechanism between MLSL and rubber the interaction energy ($\triangle E_{interaction}$) was investigated by molecular dynamics simulation. DFT

calculation was conducted at ωB97X-D/6-31g** using G09 Software [1, 2]. Consequently, the interaction energy was calculated from the following equation [3]:

$$\triangle E_{interaction} = (E_{AB} - (E_A + E_B)) \quad (1)$$

Where E_{AB} and E_A (and E_B) are the total potential energies of final system and initial system, respectively.

Note: Structure-A and B were used to represent MLSL and natural rubber segment, respectively.

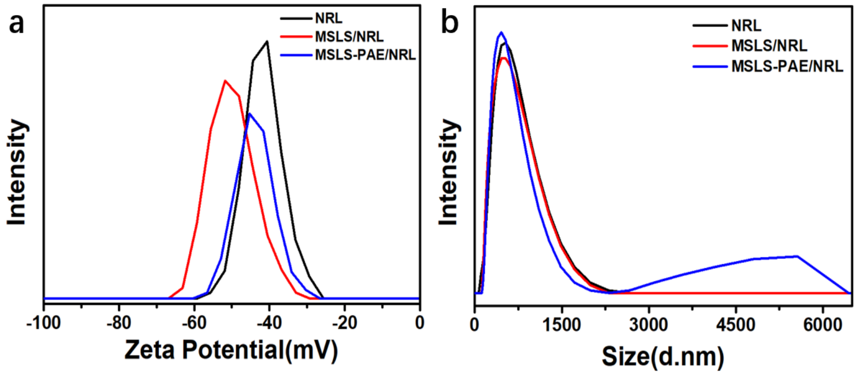

Figure S3. (**a**) Zeta potential and (**b**) particle size distribution of NRL, MSLS/NRL and MSLS-PAE/NRL.

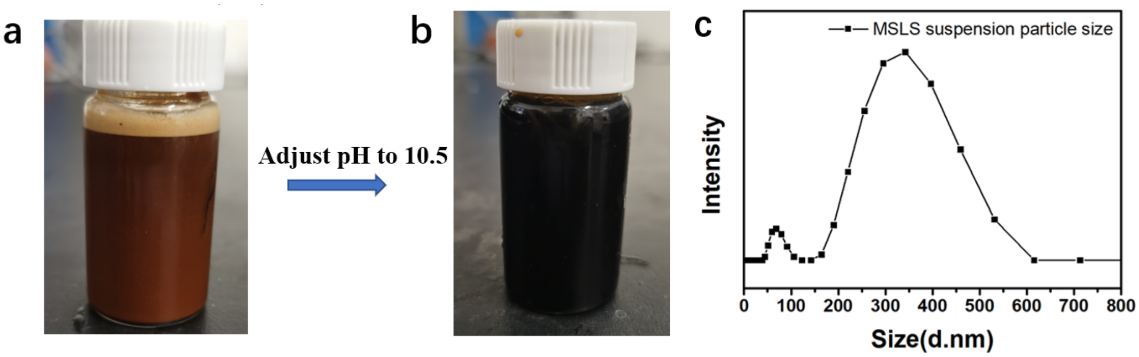

Figure S4. (**a**) During the oxidation, a picture of MSLS small brown particles, (**b**) a picture of MSLS suspension appear and the (**c**) particle size distribution of MSLS suspension.

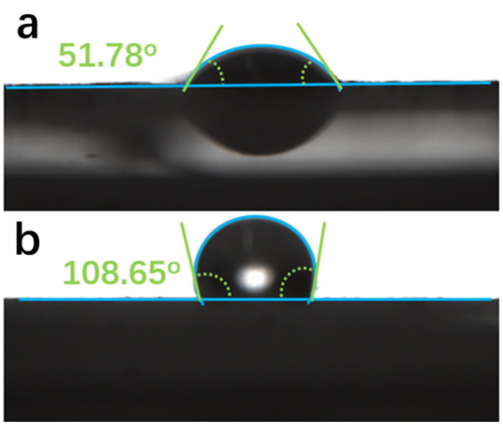

Figure S5. Water contact angle of (**a**) SLS and (**g**) MSLS.

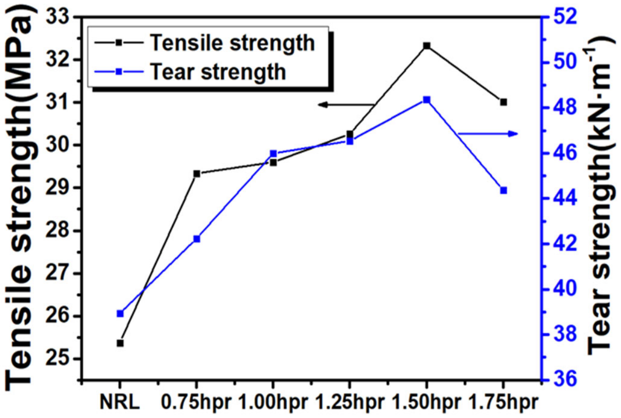

Figure S6. Tensile strength and tear strength of the composites with different MSLS content in natural rubber latex.

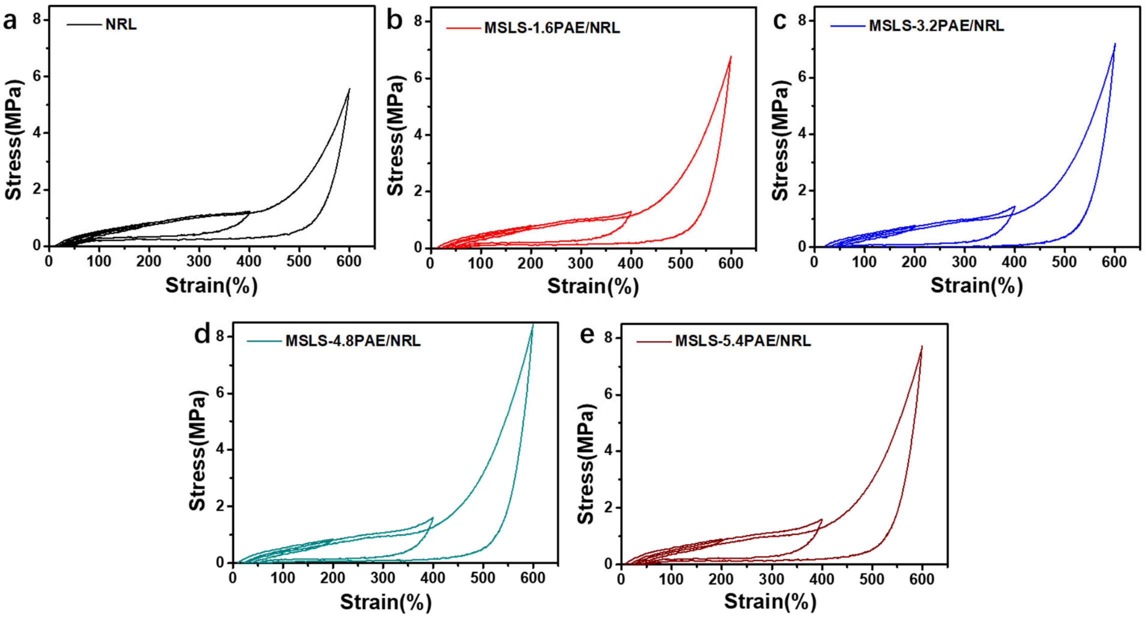

Figure S7. (**a–e**) the energy dissipation curves of rubber composites with different PAE contents.

Table S1. Zeta potential and particle size of NRL, MSLS/NRL and MSLS-PAE/NRL.

sample	Zeta Potential (mV)	Size (d. nm)
NRL	−46.37 ± 0.84	425.37 ± 8.70
MSLS/NRL	−52.90 ± 0.56	435.20 ± 17.49
MSLS-PAE/NRL	−48.57 ± 0.07	444.93 ± 5.34

Table S2. Estimated bond strength from reported references.

Bond type	Bond Energy(kJ/mol)	Ref.
C–C	370.8	[4]
C–O	344.5	[4]

C–N	338.9	[4]
S–S	267.1	[5]

Table S3. Statistics of mechanical properties of different materials.

Materials	Elongation at break (%)	Stress (MPa)	Elastic Modulus	Tear strength ($kN \cdot m^{-1}$)
NRL	771.50	25.37	3.29	41.39
MSLS-1.6PAE/NRL	761.50	32.57	4.28	48.48
MSLS-3.2PAE/NRL	809.13	34.69	4.29	55.23
MSLS-4.8PAE/NRL	884.38	37.27	4.21	61.08
MSLS-6.4PAE/NRL	789.13	33.45	4.24	57.12

References

1. Golder, M.R.; Wong, B.M.; Jasti, R. Photophysical and theoretical investigations of the cycloparaphenylene radical cation and its charge-resonance dimer. *Chem. Sci.* **2013**, *4*, 4285–4291.
2. Chai, J.D.; Head-Gordon, M. Systematic optimization of long-range corrected hybrid density functionals. *J. Chem. Phys.* **2008**, *128*, 084106.
3. Šponer, J.; Hobza, P.; Leszczynski, J. Chapter 3—Computational Approaches to the Studies of the Interactions of Nucleic Acid Bases. In *Theoretical and Computational Chemistry*; Leszczynski, J., Ed.; Elsevier: Amsterdam, The Netherlands, 1999; Volume 8, pp. 85–117.
4. Beyer, M.K. The mechanical strength of a covalent bond calculated by density functional theory. *J. Chem. Phys.* **2000**, *112*, 7307–7312, https://doi.org/10.1063/1.481330.
5. Stern, M.D.; Tobolsky, A.V. Stress-Time-Temperature Relations in Polysulfide Rubbers. *Rubber Chem. Technol.* **1946**, *19*, 1178–1192, https://doi.org/10.5254/1.3543255.
